# Inequities in Technology Access and Digital Health Literacy Among Patients With Dermatologic Conditions: Cross-Sectional Analysis of the National Health Interview Survey

**DOI:** 10.2196/51511

**Published:** 2024-03-22

**Authors:** Danny Linggonegoro, Kathryn Williams, Madeline Hlobik, Jennifer Huang

**Affiliations:** 1 Dermatology Section Division of Immunology Boston Children's Hospital Boston, MA United States; 2 Harvard Medical School Boston, MA United States; 3 Biostatistics and Research Design Center ICCTR Boston Children's Hospital Boston, MA United States; 4 Department of Dermatology Harvard Medical School Boston, MA United States

**Keywords:** teledermatology, telemedicine, telehealth, health care research, health care disparities, digital health literacy, technology access, access, accessibility, health literacy, digital literacy, disparities, disparity, equity, inequity, inequities, dermatology, dermatological, skin, cross-sectional, survey, surveys, national, HINTS, digital divide

## Abstract

Certain sociodemographic factors are associated with low technology access and digital healthy literacy.

## Introduction

As telemedicine expands, disparities in this care format should be identified and addressed. Technology access (TA) and digital health literacy (DHL)—defined by the ability to seek and appraise health information from electronic sources—are required for patients to utilize telemedicine successfully [1]. Dermatology is well suited for telemedicine due to the ability to conduct cutaneous exams with asynchronous photographs. The increased utilization of telemedicine makes it critical to identify vulnerable populations with dermatologic needs who may be unable to fully access this modality of care. Studies have shown that certain populations are less likely to participate in teledermatology visits; however, TA and DHL rates have not been described [2]. Using the National Health Interview Survey (NHIS), we sought to identify factors associated with low levels of TA and DHL among people with dermatologic conditions [3].

## Methods

### Ethical Considerations

All NHIS respondents provided oral consent prior to participation, which was voluntary. The Instituional Review Board of the Boston Children’s Hospital reviewed and exempted this study since it does not include human subjects research as defined in federal regulations (45 CFR 46.102; IRB-P00036281).

### Study Design

Participants throughout the United States were randomly selected and queried by NHIS personnel regarding their skin conditions or those of their children. The demographic data obtained included sex, age, birth country, citizenship, income, language, and insurance. Low TA was defined by reports of access to 1 or none of the following: cell phones and internet. Low DHL was defined by reports of performing 1 or none of the following health-related technology usage behaviors: using a phone or computer to receive medical information, schedule provider appointments, fill prescriptions, email providers, look up health information, or access chat groups for health information [3]. Multivariable logistic regression was used to identify factors associated with low TA and DHL. Between-group comparisons were performed via 2-tailed *t* tests for continuous variables and Wald chi-square tests for categorical variables. Sampling weights were used to account for selection variability from the complex survey design.

## Results

In 2017, a total of 26,742 adults responded (response rate: 80.7%); 7.9% (n=2113) reported a skin issue for themselves or their children. Among respondents with skin issues, 23.3% (492/2113) reported low TA, and 66.8% (1411/2113) reported low DHL. In this population, low TA was significantly associated with older age (odds ratio [OR] 1.71, 95% CI 1.54-1.91; *P*<.001), Hispanic ethnicity (OR 2.68, 95% CI 1.56-4.60; *P*<.001), living below the poverty level (OR 1.86, 95% CI 1.14-3.04; *P*=.01), public insurance (OR 2.36, 95% CI 1.46-3.82; *P*<.001), and no insurance (OR 1.99, 95% CI 1.04-3.82; *P*=.04). These factors, male sex (OR 1.70, 95% CI 1.33-2.18; *P*<.001), and Black race (OR 1.77, 95% CI 1.08-2.91; *P*=.02) were associated with low DHL (Table 1). In the total population, these demographic factors were similarly significant; however, a non-English interview language was also associated with low TA and DHL.

**Table 1:** Multivariate model of sociodemographic factors affecting low technology access and digital health literacy among patients with dermatologic issues from the 2017 National Health Interview Survey.

Characteristics			Multivariable model of low technology access					Multivariable model of low digital health literacy		
			Odds ratio (95% CI)		*P* value		Odds ratio (95% CI)			*P* value
**Sex**										
	Female (reference)	N/A^a^		N/A		N/A			N/A	
	Male	1.29 (0.98-1.68)		.07		1.70 (1.33-2.18)			<.001	
Age (increase by 10 years)			1.71 (1.54-1.91)		<.001		1.14 (1.06-1.22)			<.001
**Race and ethnicity**										
	White non-Hispanic (reference)	N/A		N/A		N/A			N/A	
	Asian non-Hispanic	1.30 (0.49-3.41)		.60		0.46 (0.23-0.89)			.02	
	Black non-Hispanic	1.70 (0.90-3.21)		.10		1.77 (1.08-2.91)			.02	
	Other non-Hispanic	1.30 (0.49-3.03)		.66		0.68 (0.30-1.53)			.34	
	Hispanic	2.68 (1.56-4.60)		<.001		2.19 (1.30-3.68)			.003	
**Language**										
	Other (reference)	N/A		N/A		N/A			N/A	
	English only	1.29 (0.56-2.95)		.55		1.02 (0.23-4.42)			.98	
**US citizenship**										
	No (reference)	N/A		N/A		N/A			N/A	
	Yes	0.53 (0.24-1.20)		.13		0.45 (0.19-1.07)			.07	
**Poverty threshold**										
	Above poverty threshold (reference)	N/A		N/A		N/A			N/A	
	Below poverty threshold	1.86 (1.14-3.04)		.01		1.85 (1.05-3.27)			.04	
**Saw general physician in the last year**										
	Yes (reference)	N/A		N/A		N/A			N/A	
	No	0.95 (0.67-1.35)		.77		1.92 (1.42-2.59)			<.001	
**Insurance**										
	Private insurance (reference)	N/A		N/A		N/A			N/A	
	Public insurance	2.36 (1.46-3.82)		<.001		1.64 (1.07-2.53)			.03	
	Uninsured	1.99 (1.04-3.82)		.04		2.09 (1.09-4.02)			.03	
	Unknown insurance	1.10 (0.79-1.54)		.57		0.93 (0.69-1.27)			.65	

^a^N/A: not applicable.

The proportion of patients with skin issues and low TA (492/2113, 23.3%) or low DHL (1411/2113, 66.8%) was significantly smaller when compared to patients without skin issues (low TA: 6649/24,629, 27%; *P*=.001; low DHL: 19,842/26,742, 74.2%; *P*<.001).

## Discussion

We identified older age, Hispanic ethnicity, poverty, and inadequate health insurance as risk factors for low TA and DHL among people reporting dermatologic issues, highlighting the importance of paying special attention to patient populations who are vulnerable to the widening gap in telemedicine access [4-6]. Male sex and Black race were associated with low DHL but not with low TA, suggesting that while these groups may have tools to access health care information, they may not know about these resources or have difficulties with utilizing them. While our study is limited by the survey’s self-reported nature, self-perceptions of TA or DHL may be more pertinent to health care technology use than objective measures.

Our results indicate that sociodemographic factors should be considered when developing telemedicine platforms for dermatologic care. Providers and office staff should ask all patients about their TA before offering telemedicine visits, and they should be aware that even patients with phones or computers may not know how to use these devices to access health care. Dermatology clinics should have trained staff to serve people who need additional assistance in accessing web-based appointments. Trust (or lack thereof) in digital health should also be considered, particularly among historically marginalized groups. On the state or national level, funding could be allocated to build community programs that promote digital health education. As telemedicine expands, it is important that practice changes do not exacerbate existing disparities for vulnerable patients.

## Abbreviations

DHL: digital health literacy
NHIS: National Health Interview Survey
OR: odds ratio
TA: technology access
